# Pyruvate kinase M2 modification by a lipid peroxidation byproduct acrolein contributes to kidney fibrosis

**DOI:** 10.3389/fmed.2023.1151359

**Published:** 2023-03-15

**Authors:** Chin-Wei Kuo, Dong-Hao Chen, Ming-Tsun Tsai, Chih-Ching Lin, Hsiao-Wei Cheng, Yeou-Guang Tsay, Hsiang-Tsui Wang

**Affiliations:** ^1^Institute of Pharmacology, College of Medicine, National Yang Ming Chiao Tung University, Taipei, Taiwan; ^2^Molecular Medicine Program, National Yang Ming Chiao Tung University, Taipei, Taiwan; ^3^Division of Nephrology, Department of Medicine, Taipei Veterans General Hospital, Taipei, Taiwan; ^4^School of Medicine, College of Medicine, National Yang Ming Chiao Tung University, Taipei, Taiwan; ^5^Institute of Biochemistry and Molecular Biology, College of Life Science, National Yang Ming Chiao Tung University, Taipei, Taiwan; ^6^Institute of Food Safety and Health Risk Assessment, National Yang Ming Chiao Tung University, Taipei, Taiwan; ^7^Doctor Degree Program in Toxicology, Kaohsiung Medical University, Kaohsiung, Taiwan

**Keywords:** renal fibrosis, acrolein, pyruvate kinase M2, hypoxia-inducible factor 1-alpha, hydralazine, carnosine

## Abstract

Renal fibrosis is a hallmark of diabetic nephropathy (DN) and is characterized by an epithelial-to-mesenchymal transition (EMT) program and aberrant glycolysis. The underlying mechanisms of renal fibrosis are still poorly understood, and existing treatments are only marginally effective. Therefore, it is crucial to comprehend the pathophysiological mechanisms behind the development of renal fibrosis and to generate novel therapeutic approaches. Acrolein, an α-,β-unsaturated aldehyde, is endogenously produced during lipid peroxidation. Acrolein shows high reactivity with proteins to form acrolein-protein conjugates (Acr-PCs), resulting in alterations in protein function. In previous research, we found elevated levels of Acr-PCs along with kidney injuries in high-fat diet-streptozotocin (HFD-STZ)-induced DN mice. This study used a proteomic approach with an anti-Acr-PC antibody followed by liquid chromatography–tandem mass spectrometry (LC–MS/MS) analysis to identify several acrolein-modified protein targets. Among these protein targets, pyruvate kinase M2 (PKM2) was found to be modified by acrolein at Cys358, leading to the inactivation of PKM2 contributing to the pathogenesis of renal fibrosis through HIF1α accumulation, aberrant glycolysis, and upregulation of EMT in HFD-STZ-induced DN mice. Finally, PKM2 activity and renal fibrosis in DN mice can be reduced by acrolein scavengers such as hydralazine and carnosine. These results imply that acrolein-modified PKM2 contributes to renal fibrosis in the pathogenesis of DN.

## Introduction

Diabetic nephropathy (DN) is a serious health problem and one of the main causes of renal failure worldwide (1). Despite recent advancements, it is vital to find new therapies for DN (2). The excessive production and deposition of extracellular matrix proteins in glomeruli and the tubulointerstitium leads to glomerulosclerosis and interstitial fibrosis in DN (3). Tubulointerstitial fibrosis is associated with abnormal glycolysis and epithelial-to-mesenchymal transition (EMT). The primary cytokine that stimulates mesangial cells and is a potent inducer of EMT is the transforming growth factor (TGF)-β (4–6). Previous research has shown that TGF-β signaling in tubule cells of diabetic mice causes abnormal glycolysis in the EMT phenotype of the fibrotic kidney (7, 8). Due to the involvement of TGF-β1 in other biological processes, however, TGF-β1 targeted therapy is unlikely to produce an efficient antifibrotic therapy. As a result, new targets for the development of novel therapeutics to halt this most damaging process in chronic kidney diseases (CKD) have been identified through a better knowledge of the various processes through which TGF-β1 modulates fibrosis.

The crucial enzyme for glycolysis, pyruvate kinase M2 (PKM2), aggregates and can be found in different forms including monomers, dimers, or tetramers, with each having different pathological roles (9, 10). For pyruvate kinase (PK) to function as a catalytic enzyme, the tetramers must be assembled correctly (11). The important posttranslational modifications in PKM2 that control cell metabolism include cysteine oxidation. Independent research has shown that the oxidation of Cys358 (12, 13), Cys424 (13), and Cys326 (14) prevents intersubunit interactions, which lowers the formation of active tetramers. By maintaining PKM2 subunit interactions and strengthening PK activity, the small-molecule activator tetraethyl pyrophosphate (TEPP)-46 promotes the assembly of the PKM2 tetramer (15, 16), Dimeric PKM2 accumulates in the nucleus and functions as a protein kinase regardless of its PK. While PKM2 activation promotes the synthesis of tetrameric PKM2 and decreases lipopolysaccharide (LPS)-induced HIF-1α accumulation (17), the PKM2 dimer interacts directly with HIF-1α, increasing HIF-1α transactivation leading to the production of its downstream glycolytic genes (18, 19). Previous studies have demonstrated that HIF-1α accumulation, abnormal glycolysis, and the EMT program can be reversed by PKM2 activation (20). These results strongly suggest that abnormal glycolysis caused by PKM2 inactivation plays a role in renal fibrosis development.

Acrolein, an α-,β-unsaturated aldehyde, is endogenously produced during lipid peroxidation, polyamine metabolism mediated by amine oxidase, and the synthesis of myeloperoxidase (21, 22). Acrolein has been linked to diabetes and its complications, including diabetic retinopathy and diabetic neuropathy (23–25). In our past studies, acrolein was shown to be a potential therapeutic target for DN (26). The biological effect of acrolein is assumed to be caused by the presence of two reactive groups. One of these reactive groups is a carbonyl group that can generate Schiff bases with primary amines, and another is an adjacent α-,β-unsaturated bond with high electrophilic properties. Through Michael addition at the β-carbon, this bond is extremely reactive with nucleophilic cellular targets (22, 27). The interactions between acrolein and nucleophilic amino acid side chains in proteins, particularly cysteine thiol residues or the ε-amino group lysine, and the imidazole nitrogen of histidine form acrolein-protein conjugates (Acr-PCs) (27, 28). In our previous study, the formation of Acr-PCs was observed in glomeruli and renal tubules of a high-fat diet (HFD)-streptozotocin (STZ)-induced DN mouse model (26). Additionally, acrolein scavengers, including N-acetylcysteine (NAC), hydralazine, and carnosine, alleviated kidney injury induced by diabetes by reducing the synthesis of Acr-PCs.

Acrolein has been shown to modify a wide range of cysteine-containing proteins in rat airway epithelial cells, and these modifications are related to cellular toxicity (29). The detrimental effects of acrolein caused by the dysregulation of proteins are involved in proliferation and apoptosis (30). However, it is still unclear which proteins directly interact with acrolein to contribute to the pathogenesis of DN. In this study, we assessed the spectrum of acrolein protein targets in kidney tissues of HFD-STZ-induced DN mice based on two-dimensional sodium dodecyl sulfate-polyacrylamide gel electrophoresis (2D-SDS-PAGE) in combination with Western blotting and mass spectrometry. Furthermore, the underlying molecular mechanisms behind acrolein-modified protein targets were investigated.

## Materials and methods

### Animal experiments

All animal research was approved by the Institutional Animal Care and Use Committee of National Yang Ming Chiao Tung University and carried out in accordance with its guidelines for Animal Research of National Yang Ming Chiao Tung University (IACUC#1090415rr). Fifty-two male C57BL/6 mice aged 6 weeks (weight 18–22 grr) were purchased from BioLASCO Taiwan Co., Ltd. (Taipei, Taiwan) and housed in a controlled environment with free access to food and water (temperature = 22 ± 1°C; relative humidity = 60 ± 5%; 12: 12 h light–dark cycle). After a week of acclimatization, the mice (*n* = 5) in the vehicle control group were either fed a normal chow diet or high-fat diet (HFD) for 16 weeks as previously described (26). After 4 weeks, streptozotocin (STZ) (50 mg/kg body weight) was intraperitoneally injected in HFD-fed mice for 7 consecutive days. Diabetic mice (*n* = 5) with fasting blood glucose levels higher than 250 mg/dl were identified and used in the subsequent experiments 5 days after the last injection. In the DN + acrolein scavenger groups, NAC (1 g/kg/d in drinking water, *n* = 5), hydralazine (50 mg/kg/d in drinking water, *n* = 5), or carnosine (50 mg/kg/d in drinking water, *n* = 5) was given to HFD/STZ-induced DN mice (*n* = 20) for 16 weeks. In the acrolein scavenger groups, the mice received a normal diet along with acrolein scavengers as previously mentioned (*n* = 4 for each scavenger). Control mice (*n* = 5) were given a normal diet.

### Biochemical analyses

Eight hours of starvation preceded the sacrifice of the mice. Then, blood samples from cardiac puncture were drawn into heparinized syringes and centrifuged for 5 min at 12,000 rpm. We measured the plasma concentrations of uric acid (UA), blood urea nitrogen (BUN), and creatinine in an Automated Clinical Chemistry Analyzer (FUJI DRI-CHEM 4000i). Urine albumin-to-creatinine ratio was determined using an albumin-to-creatinine ratio (ACR) assay kit (BioVision, Milpitas, CA, United States) in accordance with the manufacturer’s instructions after the mice were placed in metabolic cages to collect urine for 24 h.

### Immunohistochemical analysis

Each tissue sample was cut into four cross-sections, fixed in 4% paraformaldehyde, embedded in paraffin, and placed on a slide. Antigen retrieval for IHC staining was performed by heating the sections on the slides in sodium citrate buffer (10 mM sodium citrate, 0.05% Tween 20, pH 6.0) for 10 min. Then, the following primary antibodies were incubated with the tissue slices: α-SMA (1: 300, Cell Signaling #19245); collagen I (1: 100, Abcam, ab260043), fibronectin (1:100, Abcam, ab2413). All stained slides were inspected and imaged with an Olympus BX63 fluorescence microscope (Tokyo, Japan).

### Quantitative real-time polymerase chain reaction

Following the preparation of total RNA, real-time RT–PCR analysis of the cDNA was performed as described previously (31). Below are the primer sequences used for quantitative real-time PCR. The primers (5′-3′) were ACAACCACGGCCTTCCCTACTT and CACGATTTCCCAGAGAACATGTG for interleukin-6 (IL-6); TGCAGCTGGAGAGTGTGGATCCC and TGTGCTCTGCTTGTGAGGTGCTG for IL-1β; CAACAATTCCTGGCGTTACCTTGG and GAAAGCCCTGTATTCCGTCTCCTT for transforming growth factor (TGF)-β1; CCCTCCTGGCCAACGGCATG and TCGGGGCAGCCTTGTCCCTT for tumor necrosis factor (TNF)α; and GGAGGAACCTGCCAAGTATG and TGGGAGTTGCTGTTGAAG for glyceraldehyde-3-phosphate dehydrogenase (GAPDH). The relative RNA expression was analyzed by contrasting it with a control group.

### Immunoblotting analysis

Cell lysates were prepared and evaluated as described previously (31). Briefly, after blocking with 5% nonfat milk, blots were hybridized with primary antibodies overnight at 4°C. The following antibodies were utilized for immunoblotting: α-SMA (1:1000, Cell Signaling #19245), Acr-PC antibody (1:1000, raised in house) (26), collagen I (1:1000, Abcam, ab260043), fibronectin (1:1000, Abcam, ab2413), PKM2 (1:1000, Cell Signaling #4053), HIF-1α (1:1000, Sigma # ABE279), E-cadherin (1:1000, Cell Signaling #3195), Vimentin (1:1000, Cell Signaling #5741), HXK2 (1:1000, Cell Signaling #2867, Danvers, MA, United States), and GAPDH (1:5000, Cell Signaling #5174). Immunodetection was performed using enhanced chemiluminescence (ECL) (Millipore Corporation, Billerica, MA, United States).

### Two-dimensional electrophoresis and image analysis

Kidney tissues were lysed with radioimmunoprecipitation assay (RIPA) buffer with phosphatase and protease inhibitor cocktails and then harvested by centrifugation. A bicinchoninic acid (BCA) assay kit (Pierce, Dallas, TX, United States) was used to assess protein concentrations. Approximately 50 μg of protein samples were desalted by methanol-chloroform precipitation. The desalted samples were centrifuged at 70,000 rpm at 22°C for 1 h. Isoelectric focusing (IEF) was carried out with the extracted protein on an Ettan IPGphor 3 IEF (Cytiva, Marlborough, MA, United States) using Immobiline DryStrips (pI 3–10, GE Healthcare, Chicago, IL, United States). After IEF separation, the proteins were separated in the second dimension using SDS-polyacrylamide gel electrophoresis (SDS-PAGE) and the subsequent Western blot analysis and silver staining in accordance with the manufacturer’s instructions for image analysis. UVP GelStudio imagers (UVP, Analytik Jena US, Upland, CA, United States) were used to scan 2-D gels in transmission mode.

### In-gel digestion

Proteins (50 μg) were separated by 8% SDS-PAGE and the gel was stained with Coomassie blue R250 solution. The bands were excised based on the molecular weight of PKM2 or excised from silver-stained gels. The gel pieces were distained with distaining solution [25 mM NH_4_HCO_3_, 10% Na_2_S_2_O_3_, and K_3_Fe(CN)_6_]. After washing with 25 mM NH_4_HCO_3_ and 25 mM NH_4_HCO_3_/50% acetonitrile, the distained gel pieces were dried in SpeedVic. Dried gel pieces, rehydrated in 25 mM NH_4_HCO_3_ containing trypsin, and incubated at 37°C for overnight. The supernatants were collected and the remaining peptides were extracted by the repetitive extraction with 25 mM NH_4_HCO_3_ and 25 mM NH_4_HCO_3_/50% acetonitrile (ACN). After collecting and drying the supernatants, the dried digests were stored at −20°C until use.

### Protein identification by liquid chromatography–tandem mass spectrometry analysis and informatics analyzes

The protein digests were analyzed in an Agilent 1,200 nanoflow HPLC system (Agilent Technologies, Santa Clara, CA, United States) connected to an LTQ-Orbitrap hybrid tandem mass spectrometer (ThermoFisher, Waltham, MA, United States). For the setting of LTQ-Orbitrap, multiple selective ion monitoring events with Orbitrap collecting various overlapping range of m/z was set up specifically, 300–800, 625–1,075, 925–1,375, 1,225–1,675, and 1,525–1975 m/z. Full scans with Orbitrap analyzes were collected in the range of 200 to 2000 m/z as the last event. To identify various peptides, the Dynamic Exclusion function in Data Dependent Settings was enabled, with the Repeat Count set to 2, Exclusion Duration set to 180 s, and Exclusion list Size set to 50 signals. Only charge 2 and charge 3 ions were not rejected after Charge State Rejection was enabled. The top three ions in the survey scan that fulfill the above criteria were analyzed for the MS/MS with LTQ collision cell/mass analyzer. File Converter in Xcalibur SR 2.0 (TheroFisher, United States) and in-house programs written in Visual Basic for Application (VBA) were used to extract the MS/MS and calculate the charge and masses of the precursor ions. All MS/MS data were utilized in subsequent searches by the TurboSEQUEST program (Thermo Electron) using the UniProt protein sequence database for mouse proteins.

### Cell culture

A human proximal tubular epithelial cell line (HK2) was purchased from the American Type Culture Collection (ATCC, Manassas, VA, United States) and cultured in keratinocyte-serum-free medium (SFM) supplemented with 16.8 mg/mL bovine pituitary extract and 0.0347 μg/ml human recombinant EGF. For the acrolein treatments, cells were exposed with acrolein (0–10 μM) for 48 h using freshly prepared acrolein stock solution (Sigma-Aldrich, Burlington, MA, United States).

### Pyruvate kinase activity assay

Pyruvate kinase activity was assessed using a Pyruvate Kinase Assay Kit (ab83432) (Abcam, Waltham, MA, United States) according to the manufacturer’s instructions. Kidney lysates (50 μg), cell lysates collected from acrolein-treated HK2 cells (50 μg), or 5 μL of acrolein-treated pyruvate kinase (Roche, Basel, Switzerland; equivalent to 0.5 mU) were each used for the analysis of PK activity. The relative PK activity was estimated using a pyruvate standard curve and displayed in mU/mL in relation to a control.

### PKM2 crosslinking experiment

The cross-linking procedure was performed as previously described (20). In brief, kidney tissues were washed twice with PBS and incubated in 250 μM disuccinimidyl suberate (DSS; Thermo Scientific) for 30 min at room temperature. Proteins (50 μg) were lysed in 2X Bolt LDS sample buffer (Invitrogen) and boiled for 5 min at 95\u00B0C before loading to 8% SDS-polyacrylamide gel electrophoresis and followed by Western blot analysis.

### Statistical analyzes

Descriptive statistics are presented as the mean ± standard deviation (SD) or as the number (percentage). Mann–Whitney tests or Kruskal-Wallis tests were used to establish statistical significance. All calculated *p* values were two-tailed. Statistical significance was defined as *p* < 0.05. All analyzes were carried out using the IBM SPSS Statistics software package (version 23.0).

## Results

### Characterization of acrolein protein targets in kidney tissues of HFD-STZ-induced DN mice using 2D-SDS-PAGE in combination with Western blotting and mass spectrometry

**Figure 1:**
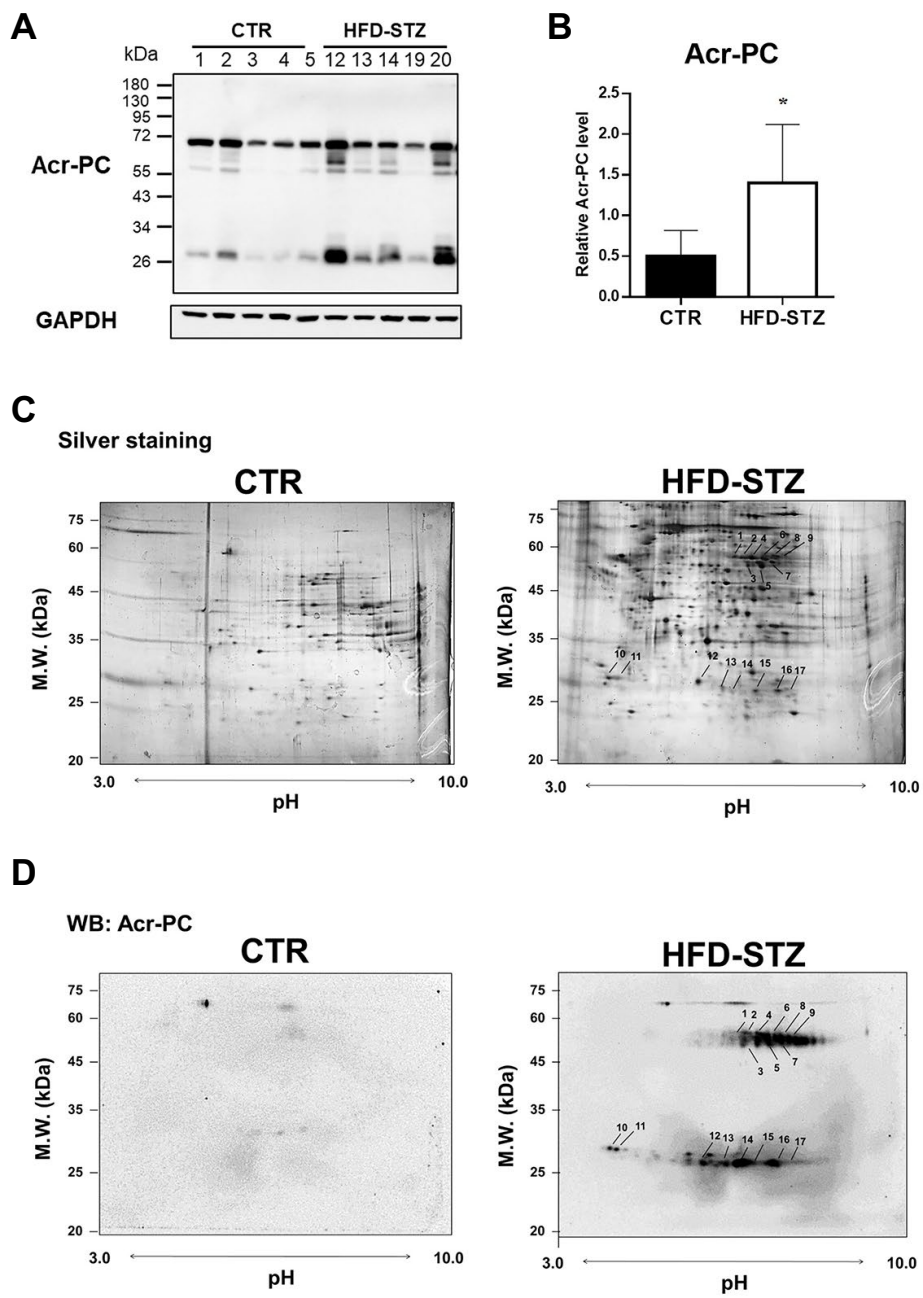
2D-SDS-PAGE in combination with Western blotting in kidney tissues of HFD-STZ-induced DN mice and vehicle control mice. Male 6-week-old C57BL/6 J mice were fed a normal diet or a high-fat diet (HFD) for 16 weeks. HFD-fed mice (*n* = 5) were treated with freshly prepared STZ (50 mg/kg/d, i.p.) for seven consecutive days, followed by continued HFD feeding for an additional 12 weeks. **(A)** Western blot analysis of Acr-PC levels in HFD-STZ-induced DN mice (HFD-STZ, *n* = 5) and vehicle control mice (CTR, *n* = 5). **(B)** Quantification of **(A)**. The values are presented as the mean ± standard deviation (SD). Mann–Whitney tests were used to determine statistical significance, and two-tailed *p* values are shown. ^*^p < 0.05 compared with the control group. **(C,D)** A representative 2-D PAGE image of kidney tissues from HFD-STZ-induced DN mice (#20) and vehicle control mice (#1) was visualized by **(C)** silver staining and **(D)** Western blot analysis. All the identified spots were localized in the perfusion index (pI) 3–10 range with a molecular mass range of 20–75 kDa. Seventeen protein spots were significantly increased in HFD-STZ-induced DN mice compared to vehicle control mice based on Western blot 2D gel patterns. These corresponding spots on silver-stained 2D gel were excised from the gel and incubated with trypsin to digest the proteins in the gel, which were then analyzed by LC–MS/MS. Protein spots identified by LC–MS/MS (arrow) are marked by their spot numbers.

The strong electrophile acrolein reacts with proteins to form Acr-PCs, resulting in alterations in protein function (22). We have previously reported that the formation of Acr-PCs is aided by the development of DN (26). However, the direct protein targets of acrolein involved in the pathogenesis of DN are still unknown. First, the biochemical analyzes of high-fat-diet-streptozotocin (HFD-STZ)-induced DN mice in comparison to control mice are shown in Supplementary Table S1. Western blot analysis was used to confirm elevated Acr-PC levels in the kidney tissues of HFD-STZ-induced DN mice (Figures 1A,B). Furthermore, a proteomics approach in conjunction with Western blotting was used to determine the direct protein targets in kidney tissues of HFD-STZ-induced DN mice. The protein extracts were prepared by differential centrifugation and then separated using 2-D electrophoresis on three replicate gels for each treatment. Silver staining (Figure 1C) and Western blot analysis (Figure 1D) were performed to visualize a representative image of the proteomic profile in HFD-STZ-induced DN mice and control mice. All identified spots ranged in molecular weight from 20 to 75 kDa and were all localized between pH values of 3 and 10. Western blotting 2D gel patterns from HFD-STZ-induced DN mice and vehicle control mice were analyzed, and the results revealed that 17 spots were substantially increased in the DN mice (the right panel of Figure 1D). These corresponding spots on the silver-stained 2D gel were identified based on the Western blot 2D gel (the right panel of Figure 1C). Trypsin was used to digest the proteins in these spots after they were excised from the gel, and the proteins were then analyzed by LC–MS/MS. The results are summarized in Table 1. These proteins included those involved in the TCA cycle, glycolysis, and redox signaling. Among these protein targets, PKM2 is the key enzyme in glycolysis and plays an important role in the pathogenesis of renal fibrosis (20, 32–34). As a result, we chose to use PKM2 for our subsequent studies.

**Table 1:** List of acrolein-modified proteins in kidney tissues of HFD mice identified by LC/MS/MS analysis.

No.	Protein name	Abbreviation	Amino acid sequence	pI/ MW (Da)
1	Carboxylesterase 1F	Ces1f	LGGASSFDGR	6.0/ 59768.9
2	Carboxylesterase 1D	Ces1d	DGASEEETNLSK	6.2/ 59839.4
3	Dihydrolipoyl dehydrogenase, mitochondrial	Dld	ADGSTQVIDTK	6.4/ 50242.7
4	Propionyl-CoA carboxylase beta chain, mitochondrial	Pccb	SVTNEDVTQEQLGGAK	6.5/ 55600.6
5	Glutamate dehydrogenase 1, mitochondrial	Glud1	NLNHVSYGR	6.7/ 55973.4
6	Methylmalonate-semialdehyde dehydrogenase [acylating], mitochondrial	Aldh6a1	VCNLIDSGTK+SSFRGDTNFYGK	7.0/ 54605.8
7	Succinyl-CoA:3-ketoacid coenzyme A transferase 1, mitochondrial	Oxct1	AGNVIFR	7.0/ 51843.9
8	Pyruvate kinase PKM	Pkm	GSGTAEVELKK	7.2/ 57808.0
9	Catalase	Cat	RFNSANEDNVTQVR	7.7/ 59626.6
10	14–3-3 protein zeta/delta	Ywhaz	VVSSIEQKTEGAEK	4.7/ 27771.1
11	Tyrosine 3-monooxygenase/tryptophan 5-monooxygenaseactivation protein, beta polypeptide	Ywhab	AVTEQGHELSNEER	4.9/ 27364.6
12	Indolethylamine N-methyltransferase	Inmt	FQHYM*VGPK	6.0/ 29440.8
13	Carbonic anhydrase 2	Ca2	IGPASQGLQK	6.5/ 28901.4
14	Bisphosphoglycerate mutase	Bpgm	MALNHGEEQVR	6.7/ 29828.6
15	Persulfide dioxygenase ETHE1, mitochondrial	Ethe1	LSGAQADLHIGEGDSIR	6.6/ 27006.7
16	Triosephosphate isomerase	Tpi1	SNVNDGVAQSTR	7.1/ 26564.7
17	Glutathione S-transferase Mu 1	Gstm1	ITQSNAILR	8.1/ 25822.0

### Acrolein adduction of PKM2 caused the suppression of PK activity, resulting in renal fibrosis

**Figure 2:**
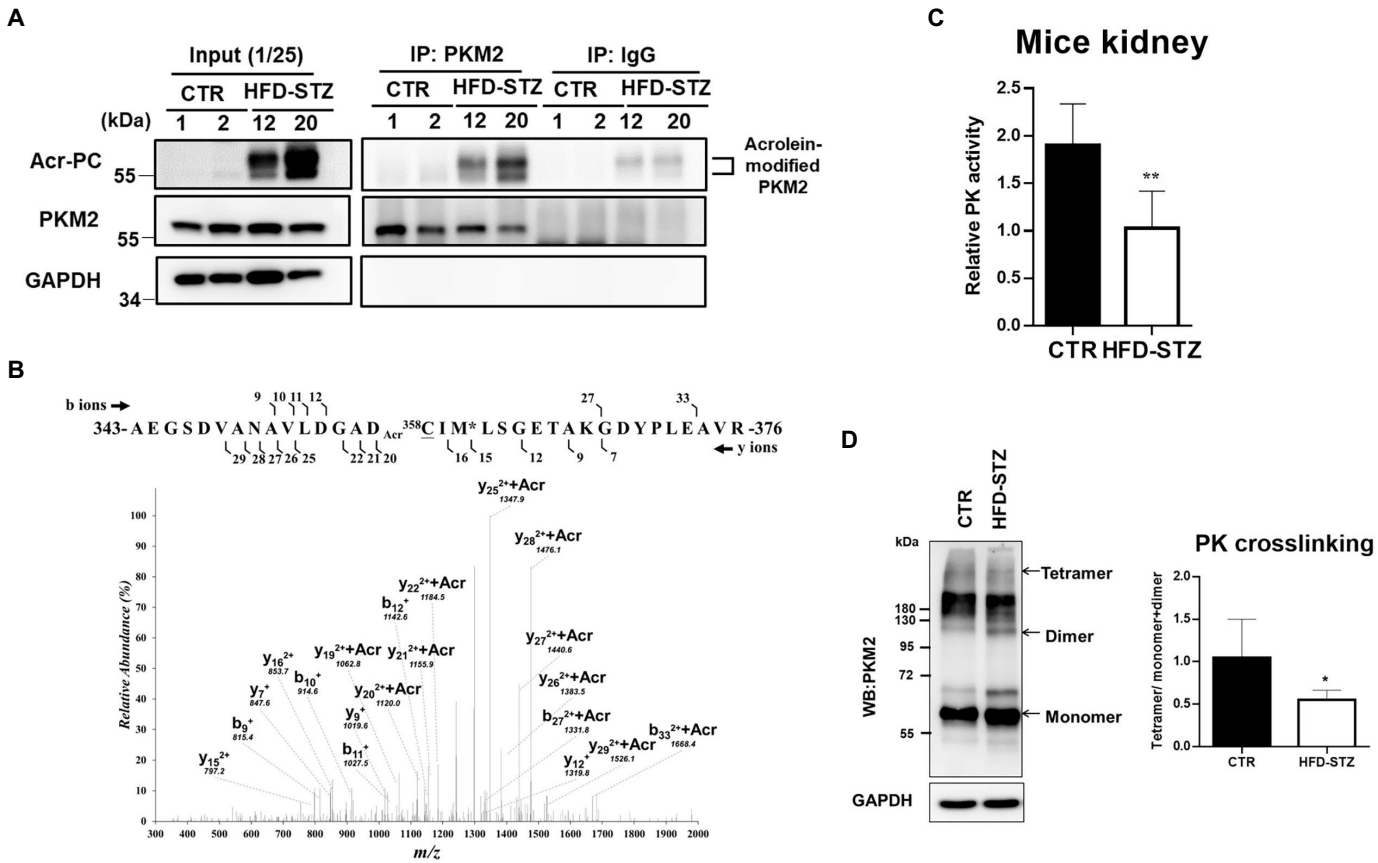
The effect of acrolein on PKM2 modification and PK activity in kidney tissues of HFD-STZ-induced DN mice. **(A)** The effect of acrolein on PKM2 was determined by using an immunoprecipitation method. Kidney lysates of HFD-STZ-induced DN mice (HFD-STZ, #12, #20) and control mice (CTR, #1, #2) were immunoprecipitated with anti-PKM2 polyclonal antibodies (IP: PKM2) or anti-rabbit IgG as a negative control, followed by immunoblotting with anti-Acr-PC, PKM2, and GAPDH antibodies. For each lysate, 40% of the quantity used for immunoprecipitation was loaded as an input control (input). **(B)** The tanden mass spectrum of acrolein-modified peptide in HFD/STZ-induced mice. The position of the peptide within the protein is indicated by the numbers of N-and C-termini of the peptide sequence. The identified b-and y-ion series are marked by the numbers above and under the peptide sequence, respectively. The putative site of acrolein modification is indicated by Acr on the front (Cys358). **(C)** Analysis of PK activity in mouse kidney samples from HFD-STZ-induced DN mice (*n* = 5) and control mice (*n* = 5). **(D)** The left panel shows a representative blot image of cross-linked mouse kidney samples showing PKM2 monomers, dimers, and tetramers. The right panel indicates the ratio between tetramer and dimer + monomer of PKM2 in kidney samples of HFD-STZ-induced DN mice (*n* = 5) and control mice (*n* = 5). The values are presented as the mean ± SD. Mann–Whitney tests were used to determine statistical significance, and two-tailed *p* values are shown. ^*^p < 0.05, ^**^p < 0.01, compared with the control group.

PKM2 was determined to be modified by acrolein in the kidney tissues of HFD-STZ-induced DN mice using an immunoprecipitation assay in conjunction with Western blot analysis (Figure 2A). The highly reactive aldehyde acrolein can react with cysteine, lysine and histidine in proteins to generate Acr-PC (27, 28). To identify the specific sites of acrolein adduction, additional LC–MS/MS analysis of kidney tissues from HFD-STZ-induced DN mice was performed, and Cys358 was revealed to be the residue most amenable to electrophilic modification (Table 2, Figure 2B). Acrolein-treated human kidney proximal cells (HK2s) also exhibited this modification (Table 2, Supplementary Figures S1A–B). PKM2 activity was decreased in kidney tissues of HFD-STZ-induced DN mice compared to control mice (Figure 2C). Acrolein also reduced the PK activity of HK2 in a concentration-dependent manner (Supplementary Figure S1C). Consistently, recombinant PK proteins were exposed to various concentrations of acrolein, and the results revealed that acrolein decreased PK activity in a dose-dependent manner (Supplementary Figure S1D). Earlier research has shown that PKM2 tetramer assembly is essential for optimal enzymatic activity (11) and that cysteine oxidation disrupts its intersubunit interaction and lowers the formation of active tetramers, which inhibits PK activity (14). A disuccinimidyl suberate (DSS)-based cross-linking study showed that HFD-STZ-induced DN mice demonstrated a lower PKM2 tetramer/dimer + monomer ratio than control mice (Figure 2D).

**Table 2:** Acrolein-modified PKM2 residues in kidney tissues of HFD mice and in acrolein-treated HK2 cells.

Z	[M + H]^+^_obs_	[M + H]^+^_cal_	△m (ppm)	Modified residues	Modified peptide
*HFD/STZ mice*					
3+	3509.649	3509.647	0.57	Cys358	^342^AEGSDVANAVLDGAD_Acr_CIM*LSGETAKGDYPLEAVR^376^
*Acrolein-treated HK2 cell*					
2+	2509.157	2509.149	3.06	Cys358	^342^AEGSDVANAVLDGAD_Acr_CIM*LSGETAK^367^

Z represents charge state, and [M + H]^+^_obs_ and [M + H]^+^_cal_ represent observed and calculated mass. The △m indicates the error calculated by observed [M + H]^+^ minus calculated [M + H]^+^ and the results divided by calculated [M + H]^+^, ppm means parts per million. Modified peptide sequences were listed as well. Acr represents acrolein modification with mass changes of 56.0262 Da.

**Figure 3:**
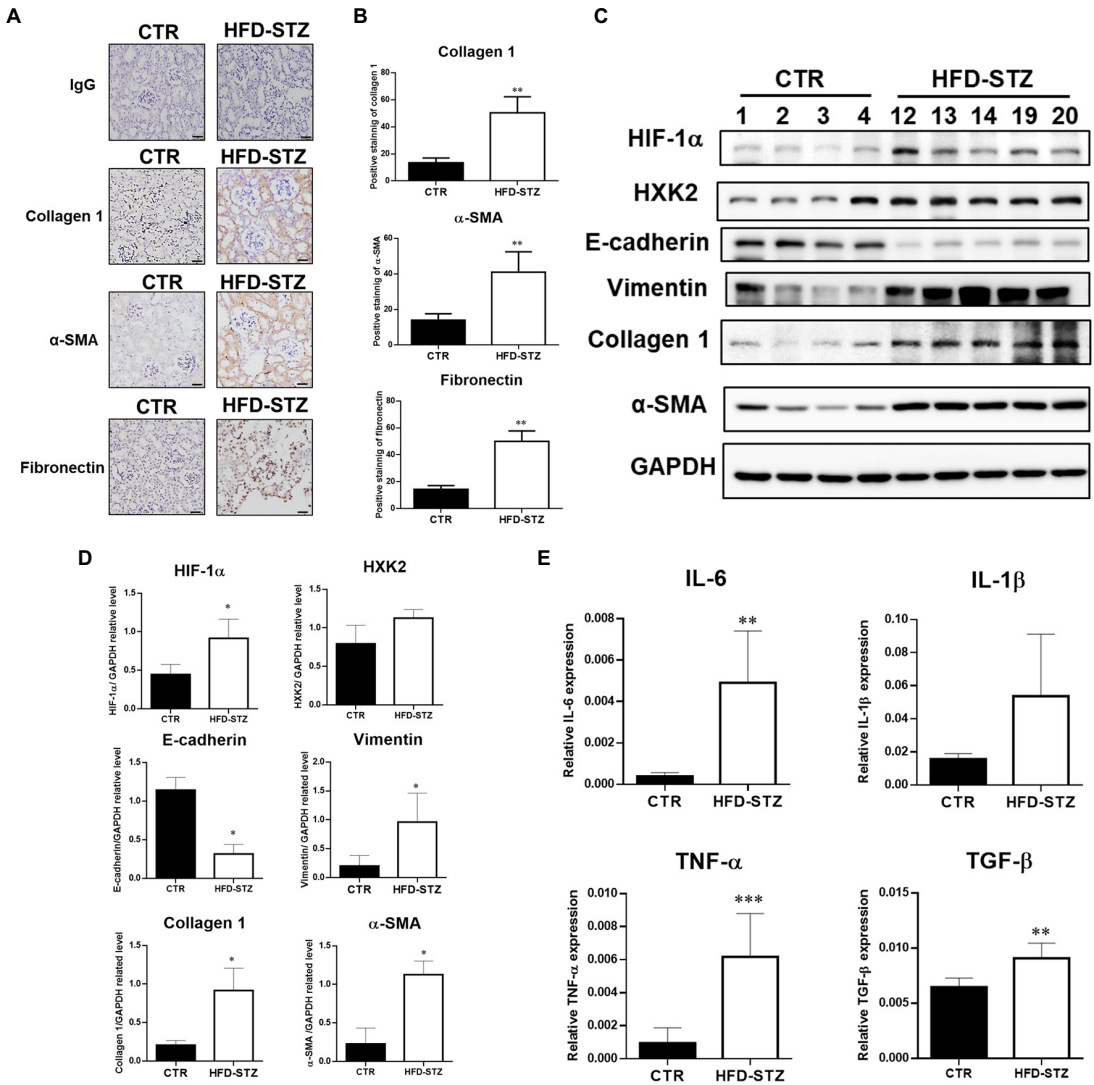
Inactivation of PKM2 is associated with aberrant glycolysis and renal fibrosis in HFD-STZ-induced DN mice. **(A)** Representative photomicrographs showed IHC staining of renal fibrosis markers, including collagen 1, fibronectin, and α-SMA, in kidney tissues in the control (*n* = 5) and HFD-STZ-induced DN mice (*n* = 5). Rabbit IgG was used as a negative control. Bars = 50 μm. **(B)** Quantification of the collagen 1-, α-SMA-or fibronectin-positive area is shown. **(C)** A representative Western blot image of HIF-1α, HXK2, E-cadherin, vimentin, collagen 1, fibronectin, or α-SMA in the control (*n* = 4) and HFD-STZ-induced DN mice (*n* = 5). **(D)** Quantification of (C). **(E)** The mRNA expression of inflammatory cytokines, including IL-6, IL-1β, TNF-α, and TGF-β in the kidney tissues of control (*n* = 5) and HFD-STZ-induced DN mice (*n* = 5) was analyzed by quantitative real-time PCR. The values are presented as the mean ± SD. Mann–Whitney tests were used to determine statistical significance, and two-tailed *p* values are shown. ^*^p < 0.05, ^**^p < 0.01, ^***^p < 0.005 compared with the control group.

According to previous studies, the inactivation of PKM2 leads to abnormal glycolysis through the activation of HIF-1α, which contributes to renal fibrosis (20). Using immunohistochemical (IHC) staining (Figures 3A,B) and Western blot analysis (Figures 3C,D), we found elevated fibrosis markers, including collagen 1, α-SMA and fibronectin, in the kidney tissues of HFD-STZ-induced DN mice. Subsequent studies on the expression of HIF-1α and downstream EMT programs, including the mesenchymal marker vimentin and the epithelial marker E-cadherin, revealed that HFD-STZ-induced DN mice showed stronger accumulation of HIF-1α and EMT activation (Figures 3C,D). Hexokinase (HXK)2 expression was higher in the HFD-STZ-induced DN mice compared to the control mice, indicating that diabetes excessively induced abnormal glycolysis (Figures 3C,D). Last, the mRNA expression of inflammatory cytokines, including interleukin-6 (IL-6), IL-1β, TNF-α, and TGF-β, was increased in the HFD-induced DN mice compared with the control mice (Figure 3E). All these results suggest that PKM2 inactivation by acrolein may contribute to renal fibrosis *via* HIF1α accumulation and induction of the EMT program and abnormal glycolysis.

### Acrolein scavengers prevented renal fibrosis linked to EMT as well as abnormal glycolysis caused by HFD-STZ

**Figure 4:**
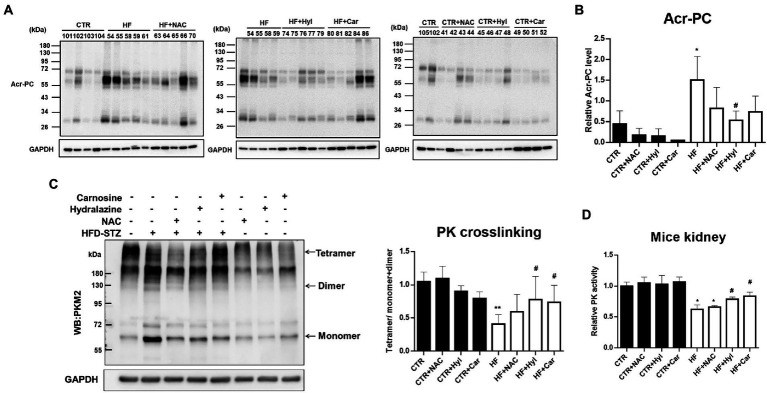
The effect of acrolein scavengers on Acr-PC formation and PKM2 activity in kidney tissues of HFD-STZ-induced DN mice. Male 6-week-old C57BL/6 J mice were fed a normal diet or a high-fat diet (HFD) for 16 weeks. In the DN group, the procedure was performed as described in Figure 1. In the DN+ acrolein scavenger group, DN mice were treated with vehicle (HF, *n* = 5) or acrolein scavengers, including NAC (HF + NAC, 1 g/kg/d in drinking water, *n* = 5), hydralazine (HF + Hyl, 50 mg/kg/d in drinking water, *n* = 5), or carnosine (HF + Car, 50 mg/kg/d in drinking water, *n* = 5) for 16 weeks. In the acrolein scavenger group, normal diet-fed mice were treated with acrolein scavengers as described above (*n* = 4 for each scavenger, CTR + NAC, CTR+ Hyl, CTR + Car). Control mice (CTR, *n* = 5) were fed a normal diet and received vehicle administration. **(A)** Western blot analysis of Acr-PC levels in kidney tissues of the control group (CTR, *n* = 5), DN group (HF, *n* = 5), DN + acrolein scavenger group (*n* = 5 for each scavenger, HF + NAC, HF + Hyl, HF + Car) and acrolein scavenger group (*n* = 4 for each scavenger, CTR + NAC, CTR + Hyl, CTR + Car). **(B)** Quantification of **(A)**. **(C)** The left panel shows a representative blot image of cross-linked mouse kidney samples showing PKM2 monomers, dimers, and tetramers. The right panel shows the ratio between tetramer and dimer + monomer of PKM2 in kidney samples of the control group (*n* = 5), DN group (*n* = 5), DN+ acrolein scavenger group and acrolein scavenger group (*n* = 5). **(D)** Analysis of PK activity in kidney tissues of the control group (*n* = 5), DN group (*n* = 5), DN+ acrolein scavenger group (*n* = 5) and acrolein scavenger group (*n* = 4). The values are presented as the mean ± SD. Kruskal-Wallis tests were used to determine statistical significance, and two-tailed *p* values are shown. ^*^p < 0.05, ^**^p < 0.01 compared with the control group. ^#^*p* < 0.05 compared with the DN group.

**Figure 5:**
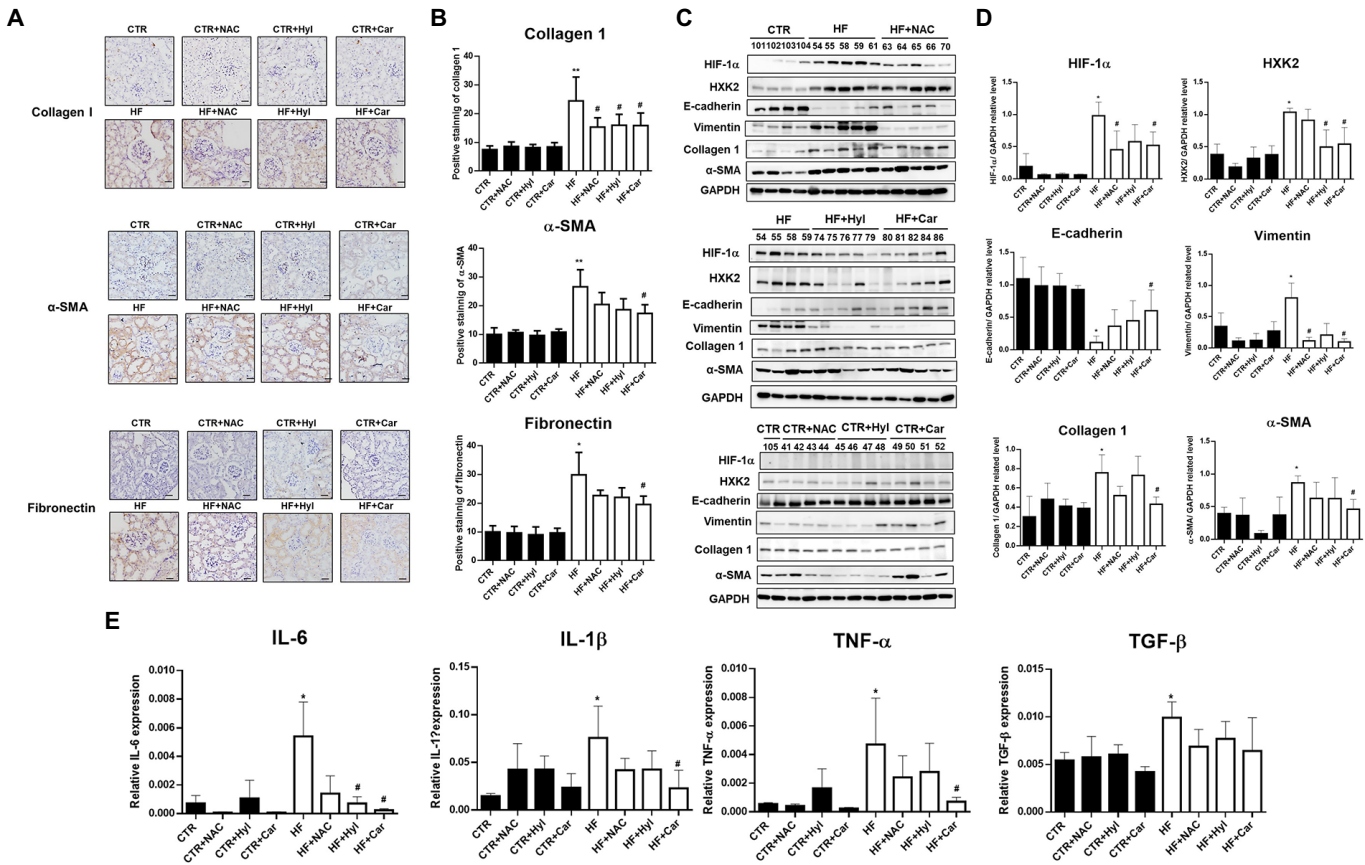
The effect of acrolein scavengers on aberrant glycolysis and renal fibrosis in kidney tissues of HFD-STZ-induced DN mice. **(A)** Representative photomicrographs showing IHC staining of renal fibrosis markers, including collagen 1, α-SMA, and fibronectin in kidney tissues in the control group (*n* = 5), DN group (*n* = 5), DN+ acrolein scavenger group and acrolein scavenger group (*n* = 5). Bars = 50 μm. **(B)** Quantification of the collagen 1-, α-SMA-or fibronectin-positive area is shown. **(C)** Western blot analysis of HIF-1α, HXK2, E-cadherin, vimentin, collagen 1, fibronectin, or α-SMA in kidney tissues of the control group (*n* = 5), DN group (*n* = 5), DN+ acrolein scavenger group and acrolein scavenger group (*n* = 5). **(D)** Quantification of (C). **(E)** The mRNA expression of inflammatory cytokines, including IL-6, IL-1β, TNF-α, and TGF-β in kidney tissues of the control group (*n* = 5), DN group (*n* = 5), DN+ acrolein scavenger group and acrolein scavenger group (*n* = 5). was analyzed by quantitative real-time PCR. The values are presented as the mean ± SD. Kruskal-Wallis tests were used to determine statistical significance, and two-tailed *p* values are shown. ^*^p < 0.05, ^**^p < 0.01 compared with the control group. ^#^*p* < 0.05 compared with the DN group.

Our earlier findings demonstrated that acrolein scavengers such as N-acetylcysteine (NAC), hydralazine (Hyl), and carnosine (Car) could lessen the kidney damage caused by diabetes (26). The biochemical analyzes of the HFD-STZ-induced DN mouse group (HF), DN + acrolein scavenger mouse groups (HF + NAC, HF + Hyl, HF + Car), and acrolein scavenger mouse groups (CTR + NAC, CTR + Hyl, CTR + Car) in comparison to control mice are shown in Supplementary Table S2. First, we used Western blot analysis to establish that these three acrolein scavengers, specifically hydralazine or carnosine, were able to reduce the formation of Acr-PC levels in kidney tissues of HFD-STZ-induced DN mice (Figures 4A,B). Compared to vehicle-treated mice, mice administered hydralazine or carnosine displayed a higher PKM2 tetramer/dimer + monomer ratio (Figure 4C). Consistently, hydralazine-or carnosine-treated mice displayed higher PK activity than vehicle-treated mice (Figure 4D). Additionally, hydralazine or carnosine was able to reduce fibrosis markers in the kidney tissues of HFD-STZ-induced DN mice according to the IHC assay (Figures 5A,B) and Western blot analysis (Figures 5C,D). The expression of HIF-1α, the downstream HXK2 and EMT program, and the mRNA expression of inflammatory cytokines were reduced in the kidney tissues of HFD-STZ-induced DN mice after treatment with hydralazine or carnosine (Figures 5C–E). These results suggest that hydralazine or carnosine reduced the expression of fibrosis markers and inflammatory cytokines in kidney tissues of HFD-STZ-induced DN mice by reducing the expression of HIF1α, abnormal glycolysis, and EMT activation (Figure 5). These results also imply that acrolein scavengers, i.e., hydralazine or carnosine, may reverse HFD-STZ-induced renal fibrosis by preventing PKM2 inactivation by acrolein.

## Discussion

Renal failure is the most frequent cause of DN, and there are currently limited strategies to halt disease progression (1). Thus, a deeper comprehension of the pathophysiology of DN would result in the development of novel therapeutic alternatives. Our earlier research demonstrated the active participation of acrolein in the pathogenesis of DN, and acrolein scavengers, including N-acetylcysteine, hydralazine, and carnosine, have renoprotective effects on DN (26). Here, we further showed that acrolein adduction on PKM2 caused a decrease in PKM2 tetramer, a reduction in PK activity, abnormal glycolysis, EMT and ultimately renal fibrosis in HFD-STZ-induced DN mice. Acrolein scavengers, including hydralazine or carnosine, could restore tetrameric PKM2 formation and PK activity and exhibited an antifibrotic effect by inhibiting abnormal glycolysis and the EMT program.

Acrolein reacts with protein residues such as side chains of cysteine, histidine, and lysine residues as well as the free N-terminal amino group by Schiff-base formation and Michael addition (28). In this study, we used a proteomic approach with an anti-Acr-PC antibody followed by LC–MS/MS analysis to identify several acrolein-modified protein targets involved in the TCA cycle, glycolysis, and redox signaling (Table 1). Previous studies have shown that acrolein is conjugated mainly with 14–3-3 protein and members of the small GTPase family in the hippocampus of an aged APP/PS1 mouse model of Alzheimer’s disease (AD) (35). In another study, the authors identified that acrolein-protein targets included a number of stress proteins, cytoskeletal proteins, and several key proteins involved in redox signaling, including thioredoxin reductase, thioredoxin, peroxiredoxins, and glutathione S-transferase π in acrolein-exposed bronchial epithelial (HBE1) cells (30). We also identified 14–3-3 protein and glutathione S-transferase in HFD-STZ-induced DN mice (Table 1). The underlying mechanisms of these acrolein-modified protein targets in the pathogenesis of DN need further investigation. In this study, we found that PKM2, the key enzyme in glycolysis, was modified and inactivated by acrolein, leading to renal fibrosis in HFD-STZ-induced DN mice (Figures 2, 3).

The thiol group of the cysteine residue is the most reactive nucleophile in proteins and is also the most likely target for acrolein (36, 37). Cysteine is frequently involved in the catalytic activity of enzymes and is found at the active sites of many proteins. Reduced cysteine residues give structural stability to proteins by preserving a suitable secondary structure; hence, the production of protein-cysteine adducts has a variety of functional ramifications. Aldose reductase (38), protein tyrosine phosphatase 1B (39) and nuclear factor-kB (NF-kB) have all been shown to become inactive as a result of cysteine residues being altered by acrolein. In this study, we discovered that acrolein modified PKM2 at Cys358 (Table 2, Figure 2B) and decreased PK activity in kidney tissues of HFD-STZ-induced DN mice (Figure 2C). Reduced PK activities were observed in acrolein-treated HK2 cells (Supplementary Figure S1C), and cysteine modification was also identified (Table 2, Supplementary Figure S1A). However, we could not exclude the possibility that acrolein may modify PKM2 at other amino acid residues, resulting in PKM2 inactivation. After exposure of recombinant PK proteins with acrolein, the results showed that acrolein decreased PK activity in a dose-dependent manner (Supplementary Figure S1D). Meanwhile, we also identified several PKM2 amino acid sites including Cys49, Cys358, Lys207, Lys270, and Lys305 which were modified by acrolein *in vitro* (Supplementary Table S3). As a result, whether acrolein-modified PKM2 at Cys358 was crucial for acrolein-induced PKM2 inactivation *in vivo* needs further investigation.

Protein cysteine residues that have undergone oxidative changes are examples of posttranslational modifications (PTMs) (40). Notably, reactive oxygen species (ROS) and cysteine residues can react to form a variety of chemical modifications including cysteine modifications (oxidations) (41, 42). Important PTMs in PKM2 that regulate cell metabolism include cysteine oxidation. Independent studies have demonstrated that the oxidation of Cys358 (12, 13), Cys424 (13) and Cys326 (14) inhibits PKM2 by preventing intersubunit interactions and reducing the formation of active tetramers. Indeed, our results showed that the PKM2 tetramer/dimer + monomer ratio was lower in HFD-STZ-induced DN mice than in control mice, which is consistent with PK activity (Figures 2C,D). This phenomenon may have resulted from Cys358 modification of PKM2 by acrolein. PKM2, the crucial critical enzyme in glycolysis, and PKM2 insufficiency result in aberrant glycolysis by activating HIF-1α, which contributes to the pathophysiology of renal fibrosis (20). In HFD-STZ-induced DN mice, HIF-1α accumulation, an increase in the EMT program, and an elevation of the renal fibrosis markers fibronectin, collagen 1, and α-SMA were all consistently observed (Figure 3). This is the first work that, to our knowledge, demonstrates how the inactivation of PKM2 by acrolein contributes to the development of renal fibrosis. Acrolein scavengers, such as those containing sulfur (thiol) and nitrogen (amino), typically work through their chemical reactivity and trapping mechanisms (43, 44). According to our previous findings, acrolein scavengers partially improved insulin intolerance and renal function in HFD-STZ-induced DN mice (26). Here, we offered more proof that acrolein scavengers may be able to partially restore PKM2 function, prevent HIF1α accumulation, halt EMT and finally lower the expression of renal fibrosis markers (Figures 4, 5). These results add to the evidence that PKM2 modified by acrolein was rendered inactive, leading to abnormal glycolysis, EMT and renal fibrosis.

## Data availability statement

The mass spectrometry proteomics data have been deposited to the ProteomeXchange Consortium via the PRIDE [1] partner repository with the dataset identifier PXD040335.

## Ethics statement

The animal study was reviewed and approved by Animal Research of National Yang Ming Chiao Tung University (IACUC#1090415rr).

## Author contributions

C-WK, Y-GT, and H-TW designed, performed research, and analyzed data. C-WK, D-HC, C-CL, M-TT, and H-WC performed the experiments and analyzed data. C-WK, and H-TW wrote the paper. All authors contributed to the article and approved the submitted version.

## Funding

This work was supported by National Health Research Institutes, Taiwan under [NHRI-EX110-11027PI, NHRI-EX111-11027PI, and NHRI-EX112-11027PI (H-TW)], Ministry of Science and Technology, Taiwan [MOST-111-2320-B-A49-018 (H-TW)], Veterans General Hospitals and University System of Taiwan Joint Research Program [VGHUST111-G1-4-1 (C-CL) and VGHUST111-G1-4-2 (H-TW)] and National Yang Ming Chiao Tung University Far Eastern Memorial Hospital Joint Research Program [#NYCU-FEMH 110DN01, 111DN01 (H-TW)].

## Conflict of interest

The authors declare that the research was conducted in the absence of any commercial or financial relationships that could be construed as a potential conflict of interest.

## Publisher’s note

All claims expressed in this article are solely those of the authors and do not necessarily represent those of their affiliated organizations, or those of the publisher, the editors and the reviewers. Any product that may be evaluated in this article, or claim that may be made by its manufacturer, is not guaranteed or endorsed by the publisher.

## Supplementary material

The Supplementary material for this article can be found online at: https://www.frontiersin.org/articles/10.3389/fmed.2023.1151359/full#supplementary-material

## Figures and Tables

**Table:** 

ACR	Albumin-to-creatinine ratio
Acr-PCs	Acrolein-protein conjugates
ACN	Acetonitrile
AD	Alzheimer’s disease
BUN	Blood urea nitrogen
CKD	Chronic kidney diseases
DN	Diabetic nephropathy
EMT	Epithelial-to-mesenchymal transition
Hyl	hydralazine
Car	Carnosine
HFD	High-fat diet
IHC	Immunohistochemical
IEF	Isoelectric focusing
LC–MS/MS	liquid chromatography–tandem mass spectrometry
LPS	lipopolysaccharide
NAC	N-acetylcysteine
PTMs	Posttranslational modifications
PKM2	Pyruvate kinase M2
ROS	Reactive oxygen species
RT-PCR	Real-time polymerase chain reaction
STZ	Streptozotocin
TEPP	Tetraethyl pyrophosphate
2D-SDS-PAGE	Two-dimensional sodium dodecyl sulfate-polyacrylamide gel electrophoresis
(TGF)-β	Transforming growth factor-β
TNFα	Tumor necrosis factor α
UA	Uric acid
