# Supplementary material for: Pyruvate kinase M2 modification by a lipid peroxidation byproduct acrolein contributes to kidney fibrosis
Source: Front Med (Lausanne). 2023 Mar 15;10:1151359. doi: 10.3389/fmed.2023.1151359 (PMC10050374; doi:10.3389/fmed.2023.1151359)
Supplement: Supplementary file 1 [file Data_Sheet_1.docx]

**Supplementary Figure**


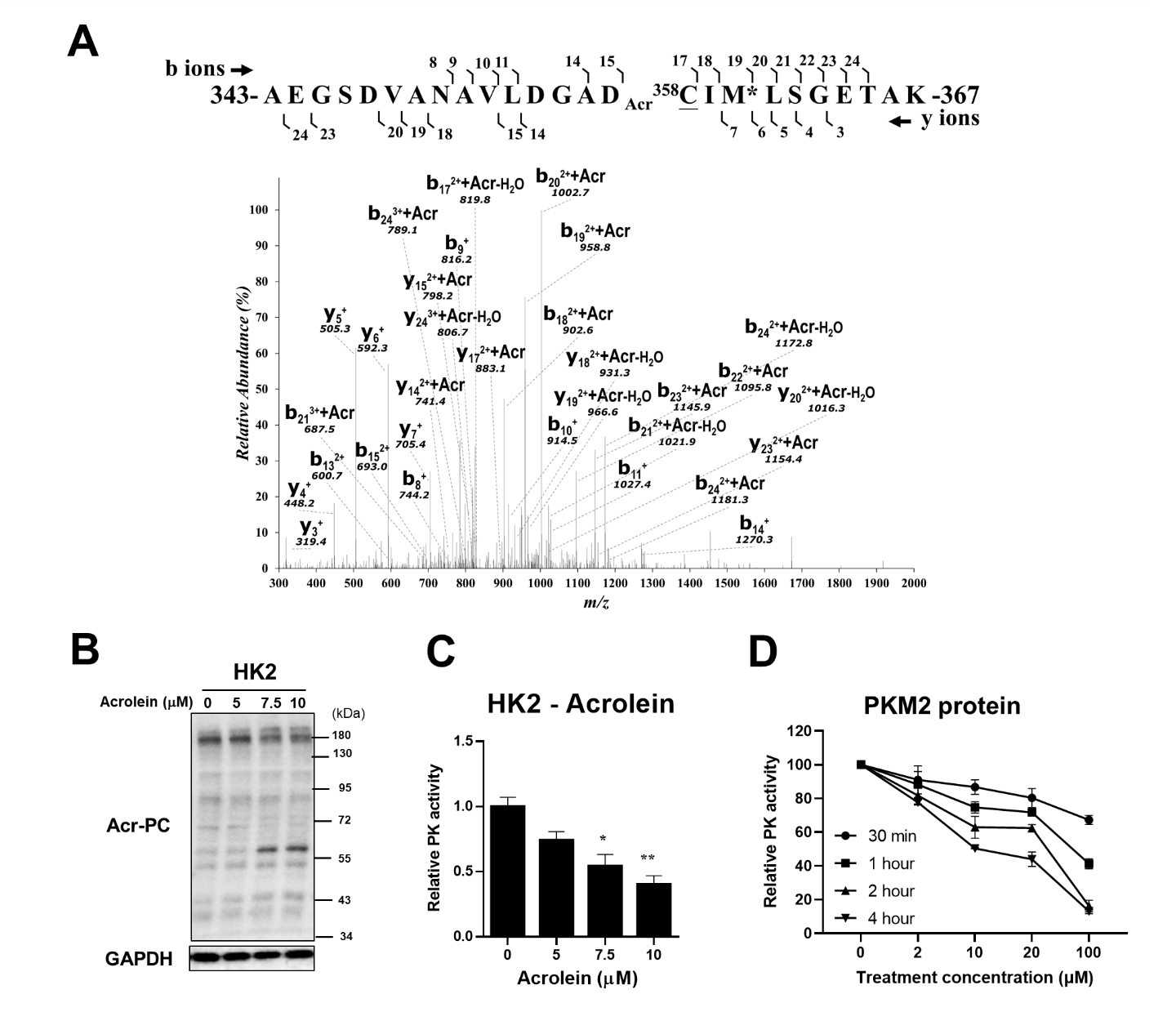


**Supplementary Figure 1. The effect of acrolein on PKM2 modification and pyruvate kinase (PK) activity in a human kidney cell line, HK2.** (A) The tanden mass spectrum of acrolein modified peptide in HFD/STZ mice. The position of the peptide within the protein is indicated by the numbers of N- and C- termini of the peptide sequence. Identified b- and y-ion series are marked by the numbers above and under the peptide sequence, respectively. The putative site of acrolein modification is indicated by Acr on the front (Cys358). (B) The expression of Acr-PC in HK2 treated with different concentrations of acrolein (0-10 μM) for 48 h was analyzed using Western blot analysis. (C) Analysis of PK activity in HK2 treated with different concentrations of acrolein (0-10 μM) for 48 h. The values are presented as the mean ±SD. Kruskal-Wallis tests were used to determine statistical significance, and two-tailed P values are shown. *P<0.05, **P<0.01 compared with the control group. (D) Analysis of PK activity in PK recombinant proteins treated with different concentrations of acrolein (0-100 μM) for 30 min to 4 h.

**Supplementary Tables**

**Supplementary Table 1. Biochemical analysis of HFD-STZ induced DN mice compared to control mice.**

|  | CTR | HFD-STZ | P value^a^ |
| --- | --- | --- | --- |
| Body weight (g) | 30.0 ± 1.2 | 30.6 ± 3.4 | 0.718 |
| Blood glucose (mg/dL ) | 129.6 ± 20.3 | 364.2 ± 95.9 | <0.005*** |
| Serum creatinine (mg/dL) | 2.0 ± 0.4 | 4.1 ± 0.9 | <0.005*** |
| Blood urea nitrogen (mg/dL) | 22.9 ± 3.5 | 29.2 ± 2.5 | 0.013* |
| Blood uric acid (mg/dL) | 2.0 ± 0.4 | 2.9 ± 0.6 | 0.032* |
| UACR (mg/g) | 108.6 ± 49.2 | 398.7 ± 103.1 | <0.005*** |
|  |  |  |  |
| The values are presented as the mean ±SD. Mann-Whitney tests were used to determine statistical significance, and two-tailed p values are shown. *p<0.05, **p<0.01, ***p<0.005 compared with control group. | | | |

**Supplementary Table 2. Biochemical analysis of HFD-STZ induced DN mice treated with acrolein scavengers including N-acetylcysteine (NAC), hydralazine (Hyl) and carnosine (Car) and vehicle control.**

|  | CTR | HF | HF+  NAC | HF+  Hyl | HF+  Car | CTR+  NAC | CTR+  Hyl | CTR+  Car |
| --- | --- | --- | --- | --- | --- | --- | --- | --- |
| Body weight (g) | 29.6±1.7 | 29.5±1.9 | 26.3±4.4 | 32.2±4.9 | 30.7±3.8 | 27.9±1.9 | 30.7±1.3 | 30.7±1.0 |
| Blood glucose (mg/dL ) | 131.6±19.1 | 404.0±45.1 ^a^ | 346.2±70.2 ^a^ | 297.2±95.2 ^a^ | 392.0±41.6 ^a^ | 115.0±19.0 | 118.5±8.3 | 128.5±14.3 |
| Compared with CTR |  | P<0.001 | P<0.001 | P<0.01 | P<0.001 |  |  |  |
| Serum creatinine (mg/dL) | 1.9±0.3 | 4.3±0.9 ^a^ | 4.4±0.1 ^a^ | 3.4±0.4 ^a^ | 3.0±0.8 ^a,b^ | 2.7±0.5 | 2.1±0.3 | 2.3±1.0 |
| Compared with CTR |  | P<0.001 | P<0.001 | P<0.001 | P<0.05 |  |  |  |
| Compared with HF |  |  |  |  | P<0.05 |  |  |  |
| Blood urea nitrogen (mg/dL) | 26.3±3.9 | 32.6± 4.3^a^ | 22.3±3.9 ^b^ | 22.1±3.9 ^b^ | 18.1±2.7 ^a,b^ | 24.9±3.5 | 26.5±2.0 | 18.7±4.2 |
| Compared with CTR |  | P<0.05 |  |  | P<0.01 |  |  |  |
| Compared with HF |  |  | P<0.01 | P<0.005 | P<0.001 |  |  |  |
| Blood uric acid (mg/dL) | 1.9±0.5 | 2.0±0.6 ^a^ | 2.7±0.5 ^a^ | 1.4±0.1 ^a,b^ | 1.8±0.6 ^b^ | 1.4±0.5 | 2.1±0.4 | 2.4±0.5 |
| Compared with CTR |  | P<0.05 | P<0.05 | P<0.05 |  |  |  |  |
| Compared with HF |  |  |  | P<0.001 | P<0.05 |  |  |  |
| UACR (mg/g) | 100.6± 47.4 | 521.2± 183.4 ^a^ | 239.3±63.9 ^a,b^ | 196.2±42.0 ^a,b^ | 277.5±124.4 ^a,b^ | 132.0±63.8 | 189.7±41.5 | 173.9±44.7 |
| Compared with CTR |  | P<0.005 | P<0.005 | P<0.005 | P<0.05 |  |  |  |
| Compared with HF |  |  | P<0.05 | P<0.005 | P<0.05 |  |  |  |
| The values are presented as the mean ±SD. Kruskal-Wallis tests were used to determine statistical significance, and two-tailed P values are shown. ^a^*P<0.05 compared with the control group. ^b^#P<0.05 compared with the DN group. | | | | | | | | |

**Supplementary Table 3. Acrolein-modified PKM2 residues in acrolein-treated PK recombinant protein.**

| **Z** | **[M+H]^+^_obs_** | **[M+H]^+^_cal_** | **△m**  **(ppm)** | **Modified Residues** | **Modified peptide** |
| --- | --- | --- | --- | --- | --- |
| 2+ | 2509.1598 | 2509.1489 | 4.34 | Cys358 | 342AEGSDVANAVLDGAD_Acr_C^+56^IM*LSGETAK367 |
| 2+ | 1358.7058 | 1358.7102 | -3.25 | Cys49 | 44NTGII_Acr_C^+56^TIGPASR56 |
| 2+ | 1841.0135 | 1841.0173 | -2.06 | Lys207 | 207_Acr_K^+76^GVNLPGAAVDLPAVSEK224 |
| 2+ | 1859.0249 | 1859.0279 | -1.59 | Lys207 | 207_Acr_K^+94^GVNLPGAAVDLPAVSEK224 |
| 3+ | 1470.8067 | 1470.8069 | -0.14 | Lys270 | 267IIS_Acr_K^+76^IENHEGVR278 |
| 2+ | 1904.046 | 1904.0533 | -3.83 | Lys305 | 295GDLGIEIPAE_Acr_K^+76^VFLAQK311 |
| PK recombinant proteins were treated with acrolein (100 μM) for 4 h followed by LC-MS/MS analysis. Z represents charge state, and [M+H]^+^_obs_ and [M+H]^+^_cal_ represent observed and calculated mass. The △m indicates the error calculated by observed [M+H]^+^ minus calculated [M+H]^+^ and the results divided by calculated [M+H]^+^, ppm means parts per million. Modified peptide sequences were listed as well. Acr represents acrolein modification with mass changes of 56.0262, 76.0313, or 94.0419 Da. | | | | | |
